# 多参数流式细胞术免疫表型分析与骨髓增生异常肿瘤诊断

**DOI:** 10.3760/cma.j.cn121090-20250729-00355

**Published:** 2025-12

**Authors:** 湘遥 肖, 艳娜 申

**Affiliations:** 1 天津医科大学医学技术学部，天津 300203 School of Medical Laboratiry, Tianjin Medical University, Tianjin 300203, China; 2 天津医科大学第二医院，天津 300211 The Second Hospital of Tianjin Medical University, Tianjin 300211, China

## Abstract

多参数流式细胞术（MFC）已被证实是骨髓增生异常肿瘤（MDS）的重要辅助诊断方法。该技术目前已在临床实践中得到普遍应用，通过简化的评分系统或精细的分析策略检测骨髓各造血细胞系的免疫表型发育异常特征，用于评估血细胞减少症及疑似MDS患者。现行指南建议将MFC分析纳入疑似MDS所致血细胞减少症的诊断评估体系。本文就该领域的现有认知与最新进展进行了综述，重点介绍以下四个方面：①样本处理与制备的优化方案；②抗体组合与荧光染料的选用标准；③骨髓细胞亚群的分析方法（包括髓系前体细胞、成熟粒细胞、单核细胞及红系造血细胞等）；④基于骨髓细胞发育异常免疫表型特征的MFC评分系统在MDS诊断中的临床应用价值。

## Abstract (en)

Multiparameter flow cytometry（MFC）has proven its value as a complementary diagnostic tool for myelodysplastic neoplasms（MDS）. It is almost universally adopted in everyday clinical practice for assessing patients with cytopenia and suspected MDS through simplified scoring systems or elaborate analytical strategies for detecting immunophenotypical dysplastic features in every hematopoietic cell lineage in the bone marrow (BM). Current guidelines recommend flow cytometric analysis as part of the diagnostic assessment of patients with cytopenia suspected of MDS. We reviewed the existing knowledge and recent advances in the field concerning ①the optimal methods of sample processing and handling, ②antibody panels and fluorochromes, ③the analysis of several BM cell subsets, such as myeloid precursors, maturing granulocytic and monocytic components, and erythropoiesis, and ④MFC scoring systems for immunophenotypic signs of myelodysplasia.

骨髓增生异常肿瘤（Myelodysplastic neoplasm，MDS），亦称骨髓增生异常综合征（Myelodysplastic syndromes，MDS），其现代诊断在骨髓和外周血涂片细胞形态学的基础上需结合细胞遗传学、免疫表型和分子学进行整合（integration）诊断[1]–[2]，尽管迄今尚无共识推荐方案，但多参数流式细胞术（Multiparameter flow cytometry，MFC）进行血细胞发育异常（dysplasia）分析已是临床MDS诊断的必检项目[3]–[5]。2009年欧洲白血病网MDS流式工作组提出过一个MDS流式细胞术（flow cytometry，FC）分析的推荐方案[6]，2014年在此基础上，国际/欧洲白血病网MDS流式工作组（ELN iMDS Flow）提出修订，并于2023年进行了更新[7]–[8]。此外，MDS国际整合创新免疫学联盟（International Integrative Innovative Immunology for MDS，i4MDS）就MDS患者的免疫细胞（T细胞、B细胞、NK细胞、树突细胞、单核/MDSC）检测分析做出了推荐[9]。本文就MFC在MDS诊断中的应用现况作一综述。

一、标本的采集与处理[7]

MFC分析标本应采用骨髓，采集量为2～3 ml，抗凝剂首选肝素，亦可用EDTA，但EDTA可能影响某些特异性抗原的表达，如CD10、CD11b、CD16和CD64，特别是在处理不及时的情况下。标本应在24 h内做处理，36 h甚至72 h内处理也可以。如果检测标本需运输，应在室温（18～25 °C）保存，尽量避免4 °C以下保存和运输。

细胞处理推荐采用染色-裂解-洗涤（stain-lyse-wash）方法，抗体染色后再进行成熟红细胞裂解，裂解液选用氯化氨。也可以采用裂解不洗涤（lysis-no-wash）方法，抗体在4 °C暗室染色后裂解成熟红细胞，然后直接上机分析。红系有核细胞成分分析在不进行成熟红细胞裂解情况下进行，结果与骨髓涂片细胞分类计数结果一致性更好。

二、单克隆抗体组套选择和流式细胞仪的推荐

抗体组套的选择应依据抗体来源、实验室使用的流式细胞仪等因素来定，不同的实验室使用的抗体组套不尽相同，ELN iMDS Flow推荐的抗体组套见表1[8]，i4MDS推荐的免疫细胞分析抗体组套见表2[9]。流式细胞仪应采用八色及以上。

**表1:** 骨髓增生异常肿瘤（MDS）流式细胞术免疫表型分析各类细胞的抗体组套（国际/欧洲白血病网MDS流式工作组推荐）

细胞类型	骨干抗体	推荐抗体	可选抗体
髓系祖细胞	CD45、CD34、CD117、HLA-DR	CD13、CD33、CD10、CD11b、CD15、CD38、CD7、CD56	TdT、CD5、CD19、CD25、CD133
淋系祖细胞	CD45、CD34	HLA-DR、CD10、CD19	CD22
粒细胞	CD45、CD117	HLA-DR、CD13、CD33、CD11b、CD16、CD10、CD15、CD14、CD64、CD56	CD34、CD5、CD7
单核细胞	CD45	HLA-DR、CD13、CD33、CD11b、CD14、CD34、CD36、CD64、CD16、CD56、CD117	CD2、MDC8（Slan）、CD300e
红细胞	CD45、CD34、CD117	HLA-DR、CD36、CD71、CD105、CD13、CD33	CD235a
其他细胞类型
嗜碱性粒细胞	CD45	CD123、HLA-DR	CD203c
肥大细胞	CD117	CD45、HLA-DR	CD2、CD25
树突状细胞	CD45、CD34、CD117	HLA-DR、CD123	CD11c、CD1c、CD141 CD303

**表2:** 免疫细胞流式细胞术抗体组套（骨髓增生异常肿瘤国际整合创新免疫学联盟推荐）

组套	免疫细胞	推荐抗体	可选抗体（免疫）	可选抗体（肿瘤）
A	T细胞	CD45、CD45RA、CD3、CD4、CD8、CD5、CD7、CD16/56、CD27、TCRγδ	HLA-DR、CD38、CD95、CD62L、CD45RO、PD1/CTLA4/TIM3	TRBC1、CD26
B	B细胞	CD45、CD20、CD19、CD10、CD27、CD38、IgD、IgM、CD25、CD22	CD305、CD185	Kappa、lambda、CD5、CD23
C	单核细胞/MDSC	CD45、CD14、CD16、CD64、CD300e、CD56、HLA-DR、CD11b、CD33、CD15、Lin	SLAN、CD141、CD45RO	CD34、CD113
D	NK细胞	CD7、CD56、CD8、CD94、CD57、CD161、CD16、CD3、KIR3DL1/DL2、CD45RO	DNAM1、NKG2D、NKp30、NKp46、KIR2DL2	
E	T细胞亚群-1	CD3、CD4、CD8、CD19、CD25、CD127、CXCR5、CXCR3、CCR4、CCR6	CD95、CD28、CCR7	
F	T细胞亚群-2	CD45RA、CD3、CD4、CD25、CD127、CD194（CCR4）、CD95、CD28、CCR7、CD8	FOXP3、CXCR5、CXCR3、CCR6、CD45RO	
G	树突状细胞	CD45、Lin、CD123、CD88、HLADR、CD5、CD11C、CD141、CD163、CD118、CD14	CD1c、CD303、CD11b、CD45RO	

**注** 每个组套关注一种特定免疫细胞亚群，利用一组核心抗体和额外抗体来查明细胞状态和功能（免疫）或明确在肿瘤背景下的失调（肿瘤）

三、各系别细胞免疫表型发育异常分析[8]

现今骨髓和外周血涂片瑞士染色光镜细胞形态学分析依然是MDS诊断分型的基石，而MFC是对细胞形态学分析的有力补充，明确规定不能单纯依据MFC分析结果来进行MDS诊断分型。MFC通过分析髓系祖细胞（Myeloid progeneitors，MP）、成熟粒细胞、单核细胞系和红细胞的抗原表达发育异常（dysplasia），作为细胞形态分析血细胞发育异常的有力补充。

1. 髓系祖细胞分析[10]：髓系祖细胞是指用CD45/SSC设门CD45^dim^/SSC^low/int^的这群细胞。分析CD34^+^细胞组分至少要分析3×10^3^个CD34^+^细胞。MDS患者造血祖细胞（Hematopoietic precursor cells，HPC）和MP（CD45^dim^SSC^low/int^ CD34^+^ CD19^-^）均常>2％，ELN iMDS Flow工作组将诊断MDS和MDS/MPN的临界值设定为3％。应同时分析CD45^dim^ SSC^low/int^CD34^+^CD19^-^和CD45^dim^/SSC^low/int^这2个细胞群的CD117表达水平，CD117表达增高常提示总生存（OS）差[8]。此外，MP异常表达CD45、CD117、HLA-DR、CD13、CD5、CD7和CD56也已证实与MDS诊断独立相关。

2. 成熟粒细胞分析[11]–[13]：成熟粒细胞与淋巴细胞比值是一个反应髓系分化成熟的指标，<1.0提示分化成熟受阻。中性粒细胞胞质颗粒减少或缺如是粒细胞发育异常形态学的一个主要特征，ELN iMDS Flow多中心研究证实成熟粒细胞与淋巴细胞的SSC比值临界值为20.1％。成熟粒细胞CD45表达减低，成熟粒细胞不同步表达祖细胞相关抗原（CD34、CD117和HLA-DR）和CD13/CD16/CD11b表达正常但CD15表达减低是粒系发育异常的主要MFC特征。此外，成熟粒细胞MFC发育异常特征还有CD33表达减低，淋系抗原（如CD5、CD7、CD19和CD56）的交叉系列表达，CD14或CD64的过表达等。

3. 单核细胞系分析[13]–[14]：单核细胞免疫表型发育异常的主要特征有：①20％～30％的MDS患者有光学散射特征减低；②16％～34％的MDS患者有单核细胞相关分化抗原（如CD11b、CD13、CD14、CD36、CD64）表达下调；③CD300e^+^成熟单核细胞比例异常减低（约18％的MDS患者）；④外周血经典型单核细胞（CD14^++^CD16^-^）≥94％对诊断慢性粒-单核细胞白血病（CMML）的敏感性和特异性分别为94.1％和92.8％。

4. 红系细胞分析[15]–[16]：红系细胞的标志有CD235a（血型糖蛋白A）和CD71（转铁蛋白受体）。红系发育异常的典型改变是CD71和CD235a的异常表达。其他红系发育异常的参数有CD36和CD71的变异系数（Coefficient of variation,CV）、CD71的平均荧光强度（Mean fluorescence intensity, MFI）、CD117^+^和CD105^+^红系细胞比例增高或减低，CD105 MFI增高或减低。

5. 免疫细胞和少见细胞分析[8]–[9]：CD34^+^细胞群中B祖细胞（CD19^+^）比例减低或完全缺乏是一个支持MDS诊断的指标。其他少见细胞如嗜酸性粒细胞、嗜碱性粒细胞、肥大细胞的MFC分析主要用于MDS的鉴别诊断，如MDS伴有系统性肥大细胞增生症、髓系和淋系肿瘤伴嗜酸性粒细胞增多症和酪氨酸激酶基因融合（Myeloid/lymphoid neoplasms with eosinophilia and tyrosine kinase gene fusions，M/LN-eo-TK）等。T淋巴细胞、NK细胞、树突状细胞、髓系衍生抑制细胞（Myeloid‐derived suppressor cells，MDSC）等免疫细胞MFC分析现阶段主要用于MDS不同疾病阶段或治疗前后的免疫状况监测，其临床意义尚待明确。此外，部分MDS患者伴有大颗粒淋巴细胞增殖（Large granular Lymphocytic proliferation，LGLP），而大颗粒淋巴细胞白血病（Large granular lymphocyte leukemia，LGLL）可表现为贫血、中性粒细胞计数减少，因此，取外周血进行T淋巴细胞和NK细胞MFC分析有助于两者的鉴别，T-LGLL的典型免疫表型为CD3^+^CD8^+^CD16^+^CD56^-^CD57^+^TCRαβ^+^（10％为TCRγδ^+^）[17]。

四、MFC积分系统与MDS诊断

由于MDS恶性克隆尚无特异性表达抗原，因此，MFC分析用于MDS诊断比较有价值的方法是采用综合不同MFC检测参数的MFC积分系统。

1. Ogata积分系统（Ogata Score）[11]–[12]：该积分系统有四个参数：CD34^+^髓系祖细胞百分比≥2％、CD34^+^细胞组分中B祖细胞比例减低（≤5％）、CD34^+^髓系祖细胞CD45表达水平（相对于淋巴细胞CD45表达水平）≤4.0％或≥7.5％和中性粒细胞侧向散射光（Sideward light scatter，SSC）与淋巴细胞参考值相比降低（≤6.0）。四项参数异常符合2项及以上则支持MDS诊断，试验组（日本队列）和验证组（意大利队列）诊断的敏感性分别为65.4％和89.4％，特异性分别为98％和90％。在该积分系统的基础上如果再加上髓系祖细胞表达CD5或CD7、或单核细胞表达CD56，则在不影响特异性的情况下可显著提高诊断敏感性。

2. RED积分系统（Red Score）[15]：该积分系统有三个参数：CD71 CV≥80，赋3分；CD36 CV≥65，赋2分；血红蛋白水平较正常值低15 g/L，赋2分。累计计分≥3分则诊断MDS，诊断敏感性为77.5％，特异性为90％，阳性预测值为97％，阴性预测值为49％。Red Score联合Ogata Score，诊断敏感性为87.9％，特异性为88.9％，阳性预测值为97％，阴性预测值为61％。

3. 整合MDS流式积分系统（integrated MDS-FC score，iFS）[18]–[19]：iFS包括44项参数，整合了Ogata Score包括的髓系祖细胞、Red Score中的红细胞系发育异常指标和FCM积分系统（FCSS）中粒细胞和单核细胞异常抗原表达指标，综合分析后再做出MDS的肯定或排除诊断。尽管iFS的诊断敏感性和特异性均优于Ogata Score和Red Score[20]，但一般实验室难以作为检测常规，新近一个多中心前瞻性研究发现联合其中17项就可以做出“支持MDS（in agreement with MDS）”的诊断结论[21]。

五、结语

MFC在临床MDS诊断中推广应用，FC分析需基于足够敏感且特异的参数、不同的实验室可重复且结果报告临床医师易懂，但由于迄今尚无MDS恶性克隆特异性抗原，MDS不是一种疾病而是一组异质性疾病，不同实验室使用的抗体组套和检测分析人员经验差别较大，因此，不能单独依据MFC分析结果来进行MDS诊断，但MFC已是MDS诊断的一个必须补充手段[22]–[24]。我国现阶段应尽量遵循ELN iMDS Flow的已有推荐，同时严格按照实验室自建检测原则来开展相关检测[25]–[26]。人工智能在FC分析中的应用，为今后多参数综合分析用于MDS诊断奠定了一定的基础，是一个非常值得关注的发展趋势[27]–[28]。
